# Playing it cool: Characterizing social play, bout termination, and candidate play signals of juvenile and infant Tibetan macaques (*Macaca thibetana*)

**DOI:** 10.24272/j.issn.2095-8137.2018.048

**Published:** 2018-05-12

**Authors:** Kaitlin R. Wright, Jessica A. Mayhew, Lori K. Sheeran, Jake A. Funkhouser, Ronald. S. Wagner, Li-Xing Sun, Jin-Hua Li

**Affiliations:** 1 Primate Behavior and Ecology Program, Central Washington University, Ellensburg Washington 98926, USA; 2 Department of Anthropology, Central Washington University, Ellensburg Washington 98926, USA; 3 Department of Biological Sciences, Central Washington University, Ellensburg Washington 98926, USA; 4 School of Resource and Environmental Engineering, Anhui University, Hefei Anhui 230601, China

**Keywords:** Social play, Play signaling, Play face, *Macaca thibetana*

## Abstract

Play behaviors and signals during playful interactions with juvenile conspecifics are important for both the social and cognitive development of young animals. The social organization of a species can also influence juvenile social play. We examined the relationships among play behaviors, candidate play signals, and play bout termination in Tibetan macaques (*Macaca thibetana*) during juvenile and infant social play to characterize the species play style. As Tibetan macaques are despotic and live in groups with strict linear dominance hierarchies and infrequent reconciliation, we predicted that play would be at risk of misinterpretation by both the individuals engaged in the play bout and by those watching, possibly leading to injury of the players. Animals living in such societies might need to frequently and clearly signal playful intent to play partners and other group members to avoid aggressive outcomes. We gathered video data on 21 individually-identified juvenile and infant macaques (one month to five years of age) from the Valley of the Wild Monkeys, Mt. Huangshan, China. We used all-occurrence sampling to record play behaviors and candidate play signals based on an ethogram. We predicted that play groups would use multiple candidate play signals in a variety of contexts and in association with the number of audience members in proximity to the players and play bout length. In the 283 playful interactions we scored, juvenile and infant macaques used multiple body and facial candidate play signals. Our data showed that juvenile and infant Tibetan macaques use a versatile repertoire of play behaviors and signals to sustain play.

## INTRODUCTION

Play is one of the most conspicuous behaviors in which animals engage. Social play combines elements of cooperation, communication, and reciprocity in participant actions. Play also incorporates behavioral modifications from other social contexts, such as agonism, mating, and predation, and thus the division between play and non-play is not always obvious (Burghardt, 2005). Although play behavior has been researched for many decades, it is variously defined depending on the field of study from which it is viewed. For example, Fagen (1981) defined play as behavior that “functions to develop, practice, or maintain physical or cognitive abilities and social relationships, including both tactics and strategies, by 2 varying, repeating, and/or recombining already functional subsequences of behavior outside their primary context” (p. 65). Fagen’s definition focuses on play function; however, even today the ultimate benefits of play remain controversial. From a structural perspective, Burghardt (2005) proposed a working definition for identifying play behaviors using five key criteria: that is, play behavior (1) has limited immediate function; (2) has an endogenous component that is voluntary, rewarding, or autotelic; (3) is structurally or temporally different from “serious” behaviors and is incomplete or exaggerated; (4) is performed repeatedly but not stereotypically; and (5) occurs when the organism is free of stress or social/physical pressures (i.e., the player is in a “relaxed field”). Burghardt (2005) further posited that these criteria must be met to label a behavior as playful in solitary or social contexts. Fagen’s definition of play behavior, coupled with Burghardt’s criteria for identifying these behaviors, offer a method to recognize and characterize play versus non-play behavior in animals of all ages (Table 1).

**Table 1:** Operational definitions

Term	Definition
Play behavior	Functions to develop, practice, or maintain physical or cognitive abilities and social relationships, including both tactics and strategies, by varying, repeating, and/or recombining already functional sub-sequences of behavior outside their primary context (Fagen, 1981, p65).
Play signal	Communicatory behaviors that function to promote, cultivate, and manage social play and demonstrate playful intentions (Bekoff, 1974; Fagen, 1981; Yanagi & Berman, 2014a, 2014b).
Successful bout	Start of the bout marked by exchange of physical contact, chasing, or other play type or play signal.

Various solitary and social play behaviors are observed across animal species (e.g., turtles, *Trionyx triunguis*: Burghardt et al., 1996; elephants, *Loxodonta Africana*: Lee & Moss, 2014; domestic dogs, *Canis familiaris*: Horowitz, 2009); however, play is particularly important to primates, with relevance to social and cognitive development (Martin & Caro, 1985; Palagi et al., 2007). Therefore, studying play can provide important insight on a species’ social relationships, how a species develops, and how they relate to cognitive abilities. Playing with juvenile and infant (hereafter: juvenile) conspecifics is typically the first non-mother activity to occur in young animals (Bekoff, 1972; Poirier, 1970). Playing with peers gradually increases in frequency, complexity, and intensity as juveniles age and their social networks expand. The repetitive modification and practice of behaviors (e.g., mounting and biting) within the play context may yield both short- and long-term benefits in future hunting, mating, or social interactions (Bekoff & Allen, 1997). The frequency of playful behavior typically declines as juveniles transition into adulthood, although adults may maintain playful relationships with juveniles (e.g., geladas, *Theropithecus gelada*: Mancini & Palagi, 2009; chimpanzees, *Pan troglodytes*: Palagi et al., 2004; Shimada & Sueur, 2014). Less frequently, adult-adult play can occur in some primate species in both sexual and non-sexual contexts (Pellis & Iwaniuk, 1999; *T. gelada*: Mancini & Palagi, 2009; bonobos, *P. paniscus*: Palagi, 2006; ring-tailed lemurs, *Lemur catta*: Palagi, 2009; Pellis & Iwaniuk, 2000; *P. troglodytes*: Yamanashi et al., 2018) but may exhibit variable forms and functions in species with different social organization. In primates, e.g., *Macaca* spp., social organization exerts a pervasive influence on a variety of behaviors, including play (Ciani et al., 2012; Fagen, 1981; Maestripieri, 2004; Thierry, 2007). Despotic species, such as Japanese macaques (*Macaca fuscata*), with high rates of aggression, low rates of reconciliation, and high levels of nepotism are characterized by a competitive, defensive, and low-risk play style (Petit et al., 2008; Reinhart et al., 2010). Additionally, mothers in despotic species may adopt a more restrictive rearing style, and mothers of low-ranking individuals may intervene when their offspring attempt to play with the offspring of high-ranking females (Maestripieri, 2004). In contrast, species that are more egalitarian and characterized by low rates of aggression and high rates of affiliation and reconciliation, such as Tonkean macaques (*Macaca tonkeana*), have a more cooperative, close contact play style and less restrictive mothers (Maestripieri, 2004; Petit et al., 2008; Reinhart et al., 2010). These play pattern differences appear to vary with social organization and exist due to the potential devolvement of play from a friendly interaction into an aggressive one that has likely negative repercussions for the players. Variability in play frequency and form also extends to the individual level, in which an individual’s “playfulness” is likely influenced by multiple factors, including personality (Lampe et al., 2017; Pellis & McKenna, 1992), prior experience (Cloutier et al., 2013), opportunity to play (Panksepp & Beatty, 1980), and neurochemistry (Siviy et al., 2011). However, much of this research is limited to human children and laboratory rats; therefore, examining individual differences in playfulness across multiple species, including primates, is necessary.

Social play fighting in juvenile animals may influence the development of dominance relationships later in life (Pellis & Pellis, 1996), and the style of play fighting may also be related to a species’ social structure and dominance style (Fagen, 1981; Palagi, 2006; Petit et al., 2008). Although gentle play fighting may be used to maintain affiliation, more intense rough-and-tumble play may establish a dominance hierarchy in post-pubertal juveniles (especially in male-male play bouts) by testing the strength of play participants (Palagi et al., 2007; Pellis & Pellis, 1996). Thus, play style may be predictive of social dominance style, and the balance between cooperation and competition within a play bout may be different depending on the nature of a species’ social system (Palagi, 2006; Petit et al., 2008; Reinhart et al., 2010). An individual may use certain behaviors, such as slap or chase, to test, cultivate, or stabilize a competitive edge in a play bout (van Leeuwen et al., 2011). By testing one’s competitive advantage against a peer during play, partners can practice aggressive interactions that may be necessary later in life to defend, maintain, or gain access to resources. Moreover, the frequency of play fighting and use of play signals in certain social systems, such as the egalitarian social structure characteristic of adult female bonobos (*P. paniscus*), may indicate the necessity of a flexible play style to assess and strengthen social relationships (Palagi, 2006).

For many primates, social play is frequently coupled with playful signals observed throughout a play bout (Fagen, 1981; Yanagi & Berman, 2014a). During play, partners transmit and receive signals, which include vocalizations (Biben & Symmes, 1986; Kipper & Todt, 2002, Vettin & Todt, 2005), body movements, gestures, and/or facial expressions (e.g., relaxed open mouth or play face; Bekoff & Allen, 1997; Pellis & Pellis, 1996). These signals may function to qualify subsequent behaviors as playful, maintain a playful context, and/or help to avoid an escalation to aggression, especially when the behaviors performed are risky or ambiguous (e.g., play bite, play slap, or play fight; Bekoff & Allen, 1997; Burghardt, 2005; Pellis & Pellis, 1996). Play signals can therefore be defined as communicatory behaviors that function to promote, cultivate, and manage social play and demonstrate playful intent (Bekoff, 1974; Fagen, 1981; Waller & Cherry, 2012; Yanagi & Berman, 2014a; Table 1). There is debate that such signals are only observed during the context of play and are distinct from the behaviors used within a play bout, and also predict the occurrence of play (Bekoff, 1974; Fagen, 1981; Yanagi & Berman, 2014a). However, many potential signals discussed in the literature (e.g., play bow in canids or play face in primates) are unclear as indicators of the beginning of a play bout; instead, these signals often punctuate the bout at different points and are variable in their duration, form, and intensity. For example, the play face, also known as the relaxed open mouth face, is a frequently-observed play signal in primates and is commonly associated with close-quarter contact, which occurs during play fights (Palagi et al., 2014; Pellis & Pellis, 1996; van Hooff, 1967). The intensity, rate, and timing of the play face can change with the intensity or behavioral content of the play interaction, thus acting as a flexible message to indicate playful intent while the dynamics of a bout quickly change (Pellis & Pellis, 1996; Špinka et al., 2016; Symons, 1978; Waller & Cherry, 2012; Waller & Dunbar, 2005). This flexibility suggests that the play face is most likely multifunctional and may also work to modulate mood (Pellis et al., 2011), invite a third party into the play bout, express playful intent to a third party, or act as a reward for both partners for playful engagement (Spijkerman et al., 1996). Play signals likely perform a critical role in prolonging play duration and maintaining multiple players in a bout (polyadic play). However, it is possible that certain factors also decrease a signal’s salience, including increase in bout length, bout intensity, misdirection or impairment of the signal, changes in audience members, or the addition of more players (Bekoff, 1972). Short play bouts may be influenced by the misinterpretation of play signals and, similarly, long play bouts with aggressive behaviors, such as wrestling, may need to include a higher frequency of intention reinforcing play signals (Spijkerman et al., 1996). Research reflects the variation and degree of frequency, flexibility, and functionality of play signals, which also appear to be influenced by species social organization. Therefore, possessing a diverse repertoire of signals (such as vocalizations and head and body movements), in addition to the iconic play face or play bow, may be advantageous for juveniles to learn and practice; one might expect multiple play signals to have evolved in any given taxon.

For many primates, social play is frequently coupled with playful signals observed throughout a play bout (Fagen, 1981; Yanagi & Berman, 2014a). During play, partners transmit and receive signals, which include vocalizations (Biben & Symmes, 1986; Kipper & Todt, 2002, Vettin & Todt, 2005), body movements, gestures, and/or facial expressions (e.g., relaxed open mouth or play face; (Bekoff & Allen, 1997; Pellis & Pellis, 1996). These signals may function to qualify subsequent behaviors as playful, maintain a playful context, and/or help to avoid an escalation to aggression, especially when the behaviors performed are risky or ambiguous (e.g., play bite, play slap, or play fight; Bekoff & Allen, 1997; Burghardt, 2005; Pellis & Pellis, 1996). Play signals can therefore be defined as communicatory behaviors that function to promote, cultivate, and manage social play and demonstrate playful intent (Bekoff, 1974; Fagen, 1981; Waller & Cherry, 2012; Yanagi & Berman, 2014a); Table 1). There is debate that such signals are only observed during the context of play and are distinct from the behaviors used within a play bout, and also predict the occurrence of play (Bekoff, 1974; Fagen, 1981; Yanagi & Berman, 2014a). However, many potential signals discussed in the literature (e.g., play bow in canids or play face in primates) are unclear as indicators of the beginning of a play bout; instead, these signals often punctuate the bout at different points and are variable in their duration, form, and intensity. For example, the play face, also known as the relaxed open mouth face, is a frequently-observed play signal in primates and is commonly associated with close-quarter contact, which occurs during play fights (Palagi et al., 2014; Pellis & Pellis, 1996; van Hooff, 1967). The intensity, rate, and timing of the play face can change with the intensity or behavioral content of the play interaction, thus acting as a flexible message to indicate playful intent while the dynamics of a bout quickly change (Pellis & Pellis, 1996; Špinka et al., 2016; Symons, 1978; Waller & Cherry, 2012; Waller & Dunbar, 2005). This flexibility suggests that the play face is most likely multifunctional and may also work to modulate mood (Pellis et al., 2011), invite a third party into the play bout, express playful intent to a third party, or act as a reward for both partners for playful engagement (Spijkerman et al., 1996). Play signals likely perform a critical role in prolonging play duration and maintaining multiple players in a bout (polyadic play). However, it is possible that certain factors also decrease a signal’s salience, including increase in bout length, bout intensity, misdirection or impairment of the signal, changes in audience members, or the addition of more players (Bekoff, 1972). Short play bouts may be influenced by the misinterpretation of play signals and, similarly, long play bouts with aggressive behaviors, such as wrestling, may need to include a higher frequency of intention reinforcing play signals (Spijkerman et al., 1996). Research reflects the variation and degree of frequency, flexibility, and functionality of play signals, which also appear to be influenced by species social organization. Therefore, possessing a diverse repertoire of signals (such as vocalizations and head and body movements), in addition to the iconic play face or play bow, may be advantageous for juveniles to learn and practice; one might expect multiple play signals to have evolved in any given taxon.

For the genus *Macaca* (23 species), visual signals during play, such as bared teeth or play face, are used differently depending on the social organizational grade of the species (Scopa & Palagi, 2016; Thierry et al., 2000). For example, bared teeth as a play signal might be held longer or performed more frequently in one species compared to another. *Macaca* species share similarities in social structure, including multi-male and multi-female groups, overlapping home ranges, and female philopatry; however, interspecific variation can be found in patterns of affiliation, reconciliation, dominance, aggression, nepotism, and temperament (Thierry, 1985, 1990; Thierry et al., 2000). Macaques are categorized on a 4-grade tolerance scale, with Grade 1 being highly hierarchical, nepotistic, and despotic (e.g., *M. mulatta* or *M. fuscata*) and Grade 4 being more tolerant or egalitarian (e.g., *M. tonkeana* or *M. nigra*) (Thierry et al., 2000). Tolerant macaque species use play signals interchangeably and redundantly to initiate and/or terminate play and self-regulate mood (Pellis et al., 2011; Scopa & Palagi, 2016). They also engage in rapid facial mimicry with their play partner, where one partner mimics the facial expression of the other (Scopa & Palagi, 2016). Such mimicry is an unconscious motor mirror response and appears to contribute to increased play duration in more tolerant species (Mancini et al., 2013; Scopa & Palagi, 2016; see Palagi & Scopa, 2017 for discussion). This flexibility in signaling may be related to an unpredictable but cooperative and less risky play style. Conversely, in despotic macaque species, ambiguous play behaviors and miscommunication likely generate increased social repercussions and physical risks; therefore, play signals may be particularly inflexible, specific, and less interchangeable in such species that exhibit high levels of aggression and competitive play style (Scopa & Palagi, 2016; Thierry et al., 2000; Yanagi & Berman, 2014b). In such cases, play signals may alert both participants and third parties that the players are “only playing,” and may be performed more often when a third party, such as a related adult or juvenile conspecific, is present and participation or interference is likely (Pellis et al., 2011).

In free-ranging despotic juvenile rhesus macaques (*M. mulatta*), play signals predict the imminent occurrence of dyadic play (Yanagi & Berman, 2014a). Furthermore, most signals are non-randomly associated with various initiations of play, indicating that signals are used selectively by the players depending upon the play content (Yanagi & Berman, 2014b). For instance, chase play is often associated with a crouch-and-stare signal, and leg-peeks are typically used by the play recipient, not the initiator (Yanagi & Berman, 2014b). For Grade 1 despotic macaques, the specific use of signaling might decrease miscommunication and help reinforce, clarify, or emphasize the playful intent of the sender, whereby indicating that participants are “only playing” alleviates potential rising tension within the group. Therefore, one might hypothesize that multiple play signals may be used in other despotic macaque species to clarify social play while mitigating potential associated aggression (Yanagi & Berman, 2014b). However, the extent to which these signals are species-specific is unknown.

Tibetan macaques (*M. thibetana*) are the largest macaque species and the most derived of their particular lineage (Fooden, 1983; Thierry, 2011). Female macaques reach adulthood at approximately four or five years of age, generally birth their first offspring between four and six years of age, and nurse infants for six to twelve months (Zhao & Deng, 1988). In males, adulthood begins at approximately seven years of age (Zhao & Deng, 1988). Tibetan macaques reproduce seasonally, and the number of offspring sired by a male is correlated with his dominance rank (Thierry, 2011; Xia et al., 2012). Tibetan macaque social organization consists of multi-male, multi-female groups of 15–50 individuals, with a female-skewed sex ratio (Berman et al., 2004; Li et al., 2007; Thierry et al., 2000; Thierry, 2011). This organization is centered on dominance hierarchies and kin-bonded coalitions (Thierry, 2011). The dominance rank of females is based on matrilines, with a daughter generally attaining the dominance rank immediately below her mother but above her older siblings (Berman et al., 2004; Thierry, 2011; Zhao, 1997). This hierarchy influences intergroup competition among females and preferential bonds between kin (Thierry, 2011). Most males disperse at adulthood and transfer between groups during their lifespan, regardless of dominance rank (Thierry, 2011; Zhao, 1997). Although group males occupy the top ranks, females can occasionally outrank males (Berman et al., 2004). Adult social relationships influence the socialization of immature individuals (Thierry, 2011), with the population generally showing a kin bias and linear hierarchies (Berman et al., 2004). As such, it is expected that the Grade 2 social organization of Tibetan macaques will affect juvenile play via third-party adult interference, whereby aggressive or affiliative behaviors are used to disrupt and terminate play. However, little is known about the play style of Tibetan macaques, as there are currently no published studies characterizing the specific play behaviors and signals observed during their playful interactions. Insight on Tibetan macaque juvenile play will provide an important foundation to examine their social and cognitive development in relation to social structure and organization.

We characterized play behaviors and identified candidate play signals in juvenile Tibetan macaques. Tibetan macaques show evidence of despotism, with a Grade 2 social organization, linear dominance hierarchies, and low conciliatory tendencies (Berman et al., 2004). It is expected that this despotic social structure will impact juvenile play behavior, play bout proximity to adults (i.e., potential third-party adult interference), frequency and distribution of play signals performed by juveniles, and composition of players within the bout (i.e., audience members). Specifically, do juveniles use specific candidate play signals in various play contexts based on the length of the bout and number of audience members in proximity? We therefore tested the following predictions: 1) due to the despotic dominance style, third-party adult interference will end play more often than other forms of play bout termination (e.g., withdrawal) (Pellis et al., 2011; Thierry, 2011; Yanagi & Berman, 2014b); 2) play behaviors and play signals will show unequal distribution at the beginning of a bout, with play signals occurring more frequently to indicate that the subsequent behaviors are playful (Yanagi & Berman, 2014a); 3) the number of play bout audience members will be positively correlated with the number of observed play signals due to an increased necessity to convey playful intent to more individuals (Bekoff, 1972; Spijkerman et al., 1996; Palagi, 2006); and 4) play duration will be positively correlated with the number of observed play signals to reinforce, clarify, and emphasize playful intent (Pellis & Pellis, 1996; Spijkerman et al., 1996; Yanagi & Berman, 2014b). We also analyzed the distribution of playful behaviors and signals across all individuals and investigated the possible effects of sex and age on the frequency of dyadic engagement in playful behaviors and play signaling.

## MATERIALS AND METHODS

### Subjects and study site

Video data were collected on 21 individually-recognized, free-ranging infant and juvenile Tibetan macaques of the Yulingkeng A1 (YA1) group located at the Valley of the Wild Monkeys in the Huangshan Scenic District, Anhui Province, China (see Berman & Li, 2002 for more information about the site). These individuals were between one month and five years old based on *Macaca* age categorization (Thierry, 2011) and current group structure maintained by the researchers of Anhui University. At the time of the study (summer 2015), 10 juvenile males were present, including (4) five year olds, (3) three year olds, (1) two year old, and (2) individuals less than 12 months old; and 11 juvenile females were present, including: (1) five year old; (3) three year olds; (4) two year olds, and (3) individuals less than 12 months old. Data collection focused on juveniles as adult-adult play is rare and may display different frequencies of play signals than that which occurs during immature play (Mancini & Palagi, 2009; Palagi et al., 2004; Shimada & Sueur, 2014). The members of YA1 have been habituated to human presence since 1986 for scientific research and since 1992 for tourism (see Berman et al., 2004). The macaques at this site are provisioned with corn three to four times daily by the park staff, and the feedings are visible to tourists (see Berman & Li, 2002). The monkeys occasionally interact with people (McCarthy et al., 2009). We obtained research approval from the Central Washington University Institutional Animal Care and Use Committee (#A021507), and our research protocols followed the legal requirements of the People’s Republic of China and the American Society of Primatologists’ Principles for the Ethical Treatment of Primates.

### Video data collection

Video data were collected at the study site from 3 August to 19 September 2015 from approximately 0800 h to 1800 h each day, resulting in 48 d in the field and approximately 400 h of video data. During preliminary data collection, inter-observer reliability in individual identification (κ=0.86) and intra-observer reliability using play behavior and signal ethograms were established (play behavior: κ=0.93, play signals: κ=0.88, bout termination: κ=1.00, actor identification: κ=0.87, and audience member identification: κ=0.86). All-occurrence sampling was used to efficiently record high quantity playful interactions (Altmann, 1974). All playful interactions defined by Fagen (1981) and outlined by Burghardt (2005) were recorded, including play behaviors (see Table 1 and Table 2) and candidate play signals (see Table 1 and Table 3), using a Canon HD Vixia camcorder. Observations were undertaken from tourist-viewing platforms and near feeding sites and other locations where juvenile and adult macaques were visible. If a second play bout occurred simultaneously while observing a preceding play bout, only play behaviors and signals in the initial bout were recorded. After the players disengaged from the play bout, recording continued until 10 s had elapsed without bout re-initiation.

**Table 2:** Definitions and components of play behaviors

Type of play	Behavior component	Definition
Chasing	Leaping, running, walking	Locomotive actions, such as running, climbing, and leaping towards or away from another individual, in which animals alternate roles of chaser and chasee, without having body contact with each other.
Cuddling	Embracing, holding, hugging, touching	Slightly resembles wrestling, but in an extremely mild form, i.e., holding each other with very slight pushing of body, but without any body displacement. Often resembles embracing.
Play biting	Biting, dragging, embracing, grabbing, hitting, leaping, lying, pinning, pulling, pushing, rolling, running, tackling, touching, walking	Play in which animals grapple and place their mouths on each other’s body. Typically involves similar behavior patterns to wrestling but occurs with biting. Biting and avoiding being bitten with body displacement are central activities.
Slapping	Hitting with hands, touching, visual fixation	Two animals hit each other with their hands for a period without proceeding to a clearer form of play, nor terminating the play encounter.
Wrestling	Dragging, embracing, grabbing, hitting, leaping, lying, pinning, pulling, pushing, rolling, running, tackling, touching, walking	Also known as rough-and-tumble play. Includes play behavior patterns in which two monkeys engage in mutual grasping, pushing, pulling, and rolling, without attempts to bite each other.

Ethogram for rhesus macaque (*Macaca mulatta*) play: Yanagi & Berman (2014a; 2014b).

**Table 3:** Definitions of play signals

Play signal	Definition
Crouch-and-stare	Animal’s ventral surface is on/near ground and its limbs are fixed, while maintaining visual fixation on partner (Symons, 1978).
Dangle-and-stare	Animal stares at partner while hanging from an object by hind limbs (Levy, 1979).
Gamboling	Bobbing, high stepping gait in which forequarters and hindquarters are alternately raised (Symons, 1978). Often accompanied by rotation of the head (Sade, 1973).
Hide-and-peek	Animal hides behind an object and then peeks at partner, alternating the two behavior patterns.
Leg-peek	Animal stares at partner through its legs with the top of its head against the ground (Symons, 1978). Animal may hold its ankles or place forearms on ground.
Look-back	Animal’s body is oriented away from partner in a fixed position on all fours, while the head is turned toward the partner over the shoulder (Levy, 1979; Symons, 1978).
Play face	Relaxed, open mouth face, typically observed during play bouts (Levy, 1979).
Roll-onto-back -and-stare	Animal rolls and lies on its back and stares at partner (Levy, 1979).
Play threat (candidate signal)	Animal directs a lunge <2 body lengths towards another individual, ending the movement by hitting the ground, without facial expression.
Slap-and-play face (candidate signal)	Animal hits another individual’s body while simultaneously directing an open mouth face towards the individual.

Adapted from Yanagi & Berman (2014b).

### Video data analysis

From the video footage, the timestamp, actor identity, audience member identity, and play signals and behaviors of the participants were coded (QuickTime Player for Mac). A modified macaque ethogram (Yanagi & Berman, 2014b) was used to analyze the play bout video data, recording all play behavior and candidate play signals observed throughout all dyadic and polyadic play bouts. Behaviors previously unlisted by Yanagi & Berman (2014b) were added to the play signal ethogram and included play threat and slap-and-play face (Table 3). We estimated each player’s proximity to group members as within arm’s reach (<50 cm) or beyond arm’s reach (>50 cm). If a group member (adult or juvenile) was in proximity to the play bout then he or she was considered part of the play bout’s audience, regardless of their orientation or activity. Each member of the play bout was counted to generate an audience member tally. If an individual performed a play signal or behavior without a group member in proximity, the audience member tally was categorized as zero. In this study, we considered play bouts “successful” when the start of the bout was marked by the exchange of contact, chasing, or other play behaviors/signals (recorded as “start of play”) (Table 1). A successful play bout was considered “terminated” when a player engaged in 1) non-play activities, e.g., grooming; 2) withdrew from the bout; or 3) adult interference occurred (Table 4; recorded as “end of play”).

**Table 4:** Definitions of play bout termination

Mode of termination	Definition
Non-play activities	Players begin to engage in any behavior/activity not considered under the category or criteria of play (Berman et al., 2004).
Withdraw	Players move out of proximity from each other (out of arms reach), and no subsequent play behaviors or signals are observed.
Adult interference	Play bout is interrupted by an adult group member performing aggressive or non-aggressive behaviors towards any player (Berman et al., 2004).

Adapted from Berman et al. (2004).

### Statistical analysis

Using IBM SPSS Statistics (Version 23), Vassar Stats Website for Statistical Computations (©Richard Lowry, 1998–2013), and UCINET (Borgatti et al., 2002), we tested each prediction with α=0.05. We only analyzed successful bouts and the signals that were displayed during such bouts. We used chi-square goodness of fit tests to assess predictions 1, 2, and 3. Spearman’s rank correlation tests were used to analyze the average number and rate (average number/min) of play signals across bout length (prediction 4). Additionally, we used chi-square goodness of fit tests to examine the distribution of the total frequency of play signals across varying audience numbers. We also used chi-squared goodness of fit tests to analyze the distribution of play behaviors and signals for each individual. Spearman’s correlation coefficient was used to test the correlation between the number of observed play signals across audience member categories. We also used MR-QAP matrix-based regression analyses in UCINET to analyze the effect of sex and age classes on the dyadic frequency of playful behaviors and signals.

While these analyses might be less robust than others, chi-square goodness of fit tests are valuable as they can use entire datasets to determine overall patterns (Reinhart et al., 2010). We used chi-squared analyses to investigate general patterns in Tibetan macaque play. However, as other authors deviate from investigations of general patterns, more complex analyses would be preferable to account for the wide variation in assumptions that are violated by play-typical data (e.g., GLMM).

## RESULTS

In total, we recorded and analyzed 283 dyadic (=2 individuals) and polyadic (≥2 individuals) play bouts, with 136 observations of the start of play, 183 observations of the termination of play, and 94 observations of a complete play bout (where the start and termination of play were clearly marked). In the 94 completed play bouts observed, average length was 64.7 s, with a range of 1 to 585 s. The number of players present in a play bout ranged from 1 to 5, and all 22 juveniles were observed to engage in play at least once.

### Distribution of play across individuals

Only positively identified individuals were included in the analyses (*n*=19). During play bouts, 16 juveniles exhibited both play behaviors and signals during bouts. We used chi-square goodness of fit tests to investigate the distribution of individual engagement in play behaviors and signals. Play behaviors were differentially (as opposed to equally) and significantly distributed across individuals (*n*=19; *χ*^2^(18)=4 016.06, *P*=0.001). Each juvenile engaged in playful behaviors between 4 and 589 times (125.35±169.58). Play signaling was also significantly and differentially (as opposed to equally) distributed across individuals (*n*=19; *χ*^2^(15)=561.52, *P*=0.001). Each juvenile exhibited play signals between 1 and 101 times (25.56±30.93). These results indicate wide variation in the frequency and use of playful behaviors and signals across individuals.

### Distribution of play across age and sex classes

We used MR-QAP regression analyses in UCINET to investigate possible correlations between the dyadic frequencies of play behaviors, play signals, age, and sex. Being of the same age class had no effect on either the dyadic frequency of playful behaviors (*Y*=–0.007*X*+0.86, *P*=0.41, *r*^2^=0.00) or play signaling (*Y*=–0.008*X*+4.53, *P*=0.43, *r*^2^<0.001). However, being of the same sex was significantly related to both the dyadic frequencies of playful behavior (*Y*=1.22*X*+0.54, *P*=0.025, *r*^2^=0.023) and play signaling (*Y*=16.91*X*+2.73, *P*=0.031, *r*^2^=0.017). Although these results showed that sex had a significant effect on play behavior and signaling, the sizes of these effects were small (*r*^2^<0.023). Given these results, we did not divide the subsequent analyses into different dyad-specific sex or age classes.

### Play bout termination

We used chi-square goodness of fit tests to compare the three play termination categories (Table 4) and test prediction 1 (third-party adult interference will end play more frequently than other forms of termination). Results showed a statistically significant difference from the expected values (*χ*^2^(2)=53.52, *P*=0.001), indicating that termination occurred more frequently due to non-playful behaviors (*n*=74) and withdrawal (*n*=94) than to third-party adult interference (*n*=16). Therefore, prediction 1 was not supported.

### Play signals

In total, 415 play signals in playful interactions were coded. The average number of play signals seen in completed play bouts (*n*=94) was 1.6 play signals per bout. We used chi-square goodness of fit tests to compare the total frequency of play bouts initiated by either a play behavior (Table 2) or a play signal (Table 3). Results showed a statistically significant difference from the expected values, and thus prediction 2 was not supported (*χ*^2^(1)=45.88, *P*=0.001): play bouts were most frequently initiated by play behaviors (*n*=108) rather than play signals (*n*=28) (an unequal distribution across bouts). In the play bouts observed (*n*=283), nine candidate play signals were recorded, including crouch-and-stare (*n*=41), dangle-and-stare (*n*=21), gamboling (*n*=3), hide-and-peek (*n*=1), look-back (*n*=1), play face (*n*=263), roll-onto-back-and-stare (*n*=19), play threat (*n*=17), and slap-and-play face (*n*=49) (Table 5). Six of the seven candidate body signals observed in rhesus macaques (Yanagi & Berman, 2014a) were also observed in Tibetan macaques (crouch-and-stare, dangle-and-stare, gamboling, hide-and-peek, look-back, and roll-onto-back-and-stare). Two additional candidate signals were also observed (play threat and slap-and-play face).

**Table 5:** Play signals observed for each audience member category

Audience number	Play face	Dangle-and-stare	Crouch-and-stare	Gamboling	Hide-and-peek	Look-back	Roll-onto-back-and-stare	Play threat	Slap-and-play face	Total (*n*)
**Zero**	0	1	8	0	0	0	5	0	0	14
**One**	84	11	18	1	1	1	7	7	7	137
**Two**	104	6	11	1	0	0	4	7	26	159
**Three**	61	3	3	1	0	0	2	1	15	86
**Four**	11	0	1	0	0	0	1	2	1	16
**Five**	3	0	0	0	0	0	0	0	0	3
**Total (*n*)**	263	21	41	3	1	1	19	17	49	415

**Figure 1:**
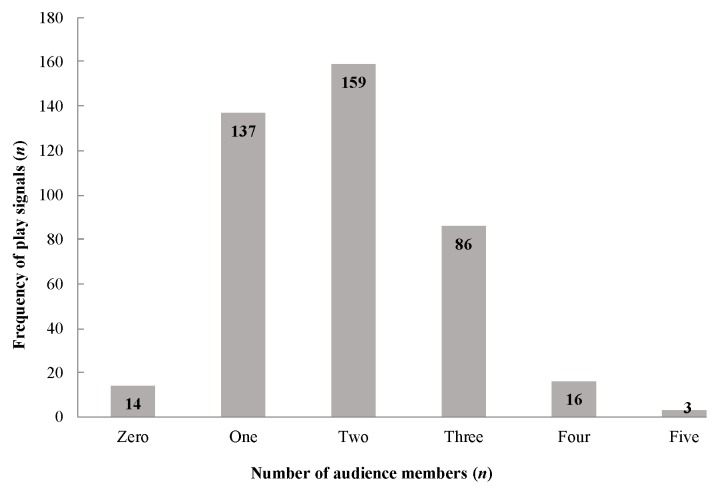
Frequency of play signals with different audience members

We compared the frequency of play signals observed for zero (*n*=14), one (*n*=137), two (*n*=159), three (*n*=86), four (*n*=16), and five audience members (*n*=3) and predicted that as the number of audience members increased, the frequency of play signals would also increase (prediction 3; Table 5). Results showed a significant statistical difference from the expected values (*χ*^2^(5)=335.46, *P*=0.001); however, the Spearman’s rank correlation between frequency of play signals and number of audience members was not significant (*r*(4)=–0.371, *P*>0.05; Figure 1). Thus, these results suggest the trend between play signals and audience members was not linear, but the frequency of play signals across audience member categories was distributed non-randomly. To examine these results further, we identified when play signals were used by a current player as a new member joined or a member withdrew from a bout. Results showed a significant deviation from the expected values (*χ*^2^(1)=6.025, *P*=0.001), and most players did not use a play signal when a new member joined the bout (no signal=230, signal=26) or when an existing member withdrew (no signal=101, signal=24).

**Figure 2:**
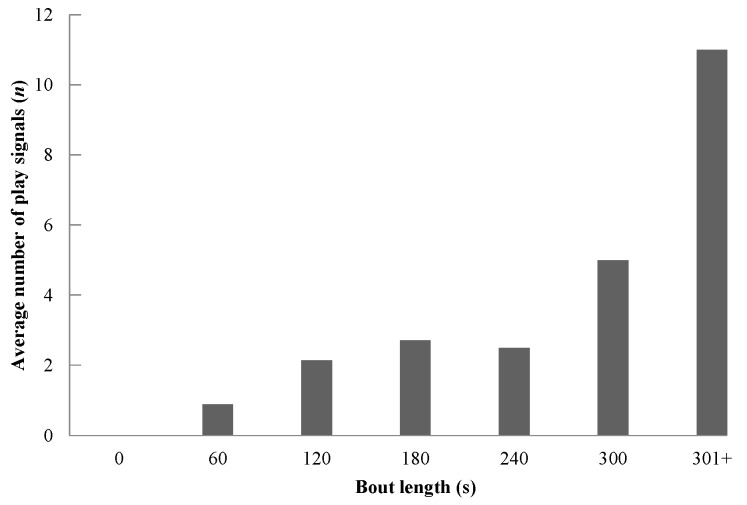
Average number of observed play signals per bout length

**Figure 3:**
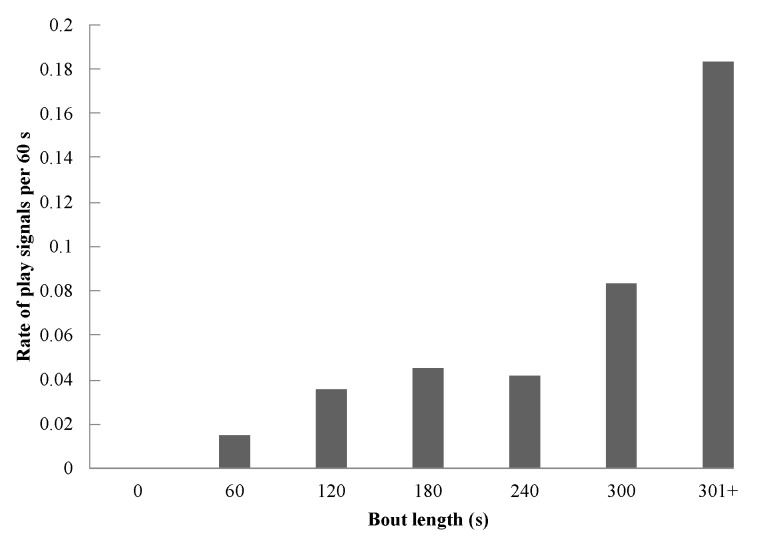
Rate of observed play signals per 60 s across bout length

We used Spearman’s rank correlation to test prediction 4, that play duration will be correlated with the rate of observed play signals. Results showed a strongly significant positive correlation between the observed rate of play signals and bout duration (Spearman’s rank correlation: *r*(5)=0.991, *P*=0.001; Figure 2) and the average number of play signals and bout duration (Spearman’s rank correlation: *r*(5)=0.964, *P*=0.001; Figure 3), thus indicating that as the length of a play bout increased, the average number and rate of play signals also increased.

## DISCUSSION

We examined the behaviors and candidate signals used in juvenile Tibetan macaque play bouts to characterize the play style of this species. Yanagi & Berman (2014b) found that juvenile rhesus macaques use play signals in selective ways, as signals were non-randomly associated with various subsequent play behaviors, and further hypothesized that signals may be necessary to reinforce and clarify playful intent. Despotic macaques may use play signals more frequently to emphasize the affiliative, rather than aggressive, nature of their behaviors. Our study showed that juvenile Tibetan macaques used similar play signals as rhesus macaques, but because these behaviors did not frequently precede the beginning of a play bout, their use may reinforce and clarify playful intent rather than signal the initiation of a bout.

Berman et al. (2004) hypothesized that due to their despotic nature, Tibetan macaque adults would interfere with play bouts perceived by third parties as aggressive. Many behaviors used in play, such as bite, wrestle, and chase, are also used in aggressive contexts and could potentially be misinterpreted as aggressive interactions. In despotic species such as Tibetan macaques, observers of play may interpret a friendly exchange as an aggressive one if it does not contain clear and frequent play signals, and consequently may terminate or disrupt the play bout. However, our results did not support this prediction. In fact, play termination in juvenile Tibetan macaques usually occurred because individuals withdrew from the bout or engaged in non-play behaviors. We observed only 16 cases in which other monkeys terminated the playful interaction: one case by a low-ranked adult male and one case by a low-ranked adult female, two cases by a mid-ranked male and five cases by a mid-ranked adult female, three cases by a high-ranked adult male and two cases by a high-ranked adult female, and two cases by a young adult female. We have insufficient data to draw conclusions regarding the identities of the individuals who terminated these play bouts, but our anecdotal evidence does not follow our prediction both in terms of the overall number of third-party terminations or the identities of the individuals who terminated the bouts. It is possible that juvenile macaques avoid areas occupied by adults to directly manage the end of play themselves and avoid adult interference (Self et al., 2013). Additionally, although prediction 1 was not supported, due to their despotic dominance style and based on the Burghardt (2005) criteria for play, young macaques may avoid adults to maintain play in a relaxed field. However, further comparative research is needed to test the hypothesis that social organization in all macaque species (including Grades 2 and 3) may influence play style (but see Reinhart et al., 2010), and therefore play termination by adult interference.

We found that more play bouts began with play behaviors than play signals (not supporting prediction 2). Therefore, play signaling in juvenile Tibetan macaques may be used similarly to signaling in juvenile rhesus macaques, in which play signals are used to clarify, reinforce, and prolong a play bout between partners (Yanagi & Berman, 2014b). Furthermore, our data showed a possible trend between the number of audience members and number of play signals generated in a bout, with more signals occurring with one or two audience members (prediction 3; Table 5; Figure 1). Several conclusions can be drawn from these results. It appears that individuals use play face (*n*=263; Supplementary Figure S1) more than other play signals (*n*=152), regardless of audience number. This indicates the salient nature of the play face signal when there is a receiver present (Pellis & Pellis, 1996; van Hooff, 1967) or when a combative play behavior, such as play slap, may escalate to aggression (Palagi et al., 2007). This was further supported by the lack of play face observed in the zero-audience member category (when no other group member was in proximity to the initiator). Therefore, play face may be an important communicative tool in polyadic play when a player is near the sender. This result suggests that play face may be a signal to others in proximity to the bout rather than generated as a signal to the players themselves (Pellis & Pellis, 1996; van Hooff, 1967). However, play face may be a general signal showing playful intention, as it can be used in a variety of contexts and to clarify play when the dynamics of the bout change (Pellis & Pellis, 1996). Moreover, the occurrence of signaling in the zero-audience member category (*n*=14) was much lower than all other categories (*n*=401), and the lack of observed play face may be an artifact of this sampling. Additionally, the frequent occurrence of play face may be an involuntary artifact of the sender’s enjoyment of the bout rather than a message for players (Spijkerman et al., 1996).

Only three play signals were observed in the zero-audience member category: (1) crouch-and-stare, (2) dangle-and-stare, and (3) roll-onto-back-and-stare. This may indicate the need to use complex body and facial signals to attract players to begin a bout, rather than a face-only signal. For example, juvenile chimpanzees often use attention-getting gestures when a play partner does not see the signal sender’s play face (Tomasello et al., 1989). In this way, a play signal that involves the combination of two or more signals may be necessary to amplify or reinforce the sender’s message. Our results support this speculation, as juvenile Tibetan macaques used multiple play signals from their diverse behavioral repertoire to indicate their willingness to play with conspecifics. However, a larger sample of zero-audience member play bouts is needed for further investigation.

Additionally, across all audience member categories, the leg-peek signal (see Table 3) was never observed. Yanagi & Berman (2014b) found that in rhesus macaques, the leg-peek was associated with play initiated by the receiver and may have served as a play invitation. However, this signal was not present in Tibetan macaque play, indicating that leg-peek may be a species-specific signal. Similarly, two candidate play signals were observed in the Tibetan macaque repertoire that were not observed in rhesus macaques: play threat (Supplementary Figure S2) and slap-and-play face (Supplementary Figure S3). Adult Tibetan macaques use threat behavior as part of an aggressive interaction, and it is characterized by an open mouth gesture and another body movement, such as a ground slap, raised eyebrow, or lunge (Berman et al., 2004). Therefore, the play threat signal, observed during play bouts, may potentially be an incomplete adult threat behavior, with the absence of the open mouth facial expression. Play threat may enable juvenile macaques to practice components of adult behavior within the context of play (Martin & Caro, 1985). However, further research is needed to confirm that the candidate play signals observed in Tibetan macaque juveniles do not occur under any other contexts. Although the characterization of Tibetan macaque play signaling differs from that of rhesus macaques, there does appear to be some cross-species similarity in both play behavior and signaling repertoires. The similarities and differences in their play style may reflect social organization (Palagi, 2006); however, more comparative research is needed.

Relative to audience members, we observed the combination of slap (a play behavior) and play face (a play signal) more than other play signals, such as gamboling, hide-and-peek, and look-back (Figure 1). It is possible that the playful intention of a slap behavior needs to be clarified as play because of its association with aggression (Burghardt, 1999; Pellis & Pellis, 1996). In this way, the slap behavior and play face signal would contradict each other and, therefore, might increase the likelihood of the receiver understanding the situation as non-threatening.

Although we expected that more play signals would be observed in polyadic play bouts (at least one actor and two audience members) (Spijkerman et al., 1996), we did not find support for this prediction. Our results indicated that two audience members (three players in total) included the largest number of play signals emitted for this bout composition. The play signals observed in play groups larger than three members were less frequent, and the number of play signals used within larger group compositions declined as more members were added. This result implies a possible threshold for the saliency of play signals past a certain audience member number. It is possible that play bouts with more players increase the complexity of the bout, making them harder to manage (Bekoff, 1972). Furthermore, the play signal message may lose salience as the complexity and size of the play group increase beyond three players. This breakdown of communication may be more readily observed in despotic macaques due to their strict social organization and therefore more risky and uncertain social interactions. It is also reasonable to conclude that play bouts with fewer members reinforce affiliative bonds between group members, whereas larger play groups may be used to assess individuals’ physical strengths (Pellis & Pellis, 1996). Therefore, signaling may be crucial in a small play group to reaffirm the social context: affiliation rather than aggression. Our results also showed that play signals were less likely to be emitted when new audience members were added to a bout or when a player left a bout. This indicates that when play signals are used by juvenile Tibetan macaques, they are used generally and in various contexts, as they were observed before, during, and when withdrawing from play (Pellis et al., 2011).

Finally, we examined the length of play bouts in relation to the number of play signals used (prediction 4; Figure 2; Figure 3). Spearman’s rank correlation showed a strong statistically significant correlation, thus supporting this prediction and indicating the likely importance of play signals in sustaining a play bout. Previous literature has shown that play bouts tend to be longer when players use the play face (Waller & Dunbar, 2005), playful facial expressions punctuate bouts rather than initiate them (Palagi et al., 2007), third parties join the play bout more often when the play face signal is used, and long play bouts are typically more intense and contain more play faces, especially when ambiguous behaviors are involved (such as wrestle and gnaw) (Spijkerman et al., 1996). This increased signaling may be critical for maintaining playful interaction, as additional evidence indicates that short play bouts are often marked by a misinterpretation of signals, thus halting play. Our study demonstrated that candidate play signals in Tibetan macaques may be used in similar ways, with the longest play bouts containing the most play signals. However, further study is needed to examine the causal relationship between play bout length and the frequency and distribution of play signals.

## CONCLUSIONS

Juvenile Tibetan macaques maintained playful interactions using multiple candidate play signals, which combined body and facial gestures, to begin play, encourage the continuation of play, and end play. Across all audience member numbers, play face was the most frequently observed play signal, possibly indicating its salient and general nature. Tibetan macaque juveniles utilized play behaviors and signals similar to rhesus macaques; however, the differences observed in their play style and signal variety may be influenced by the dominance style of Tibetan macaques.
